# Time-Dependent Oxidative Alterations in Plasma and Lung Tissue after Meconium Aspiration in a Rabbit Model

**DOI:** 10.3390/antiox12010037

**Published:** 2022-12-25

**Authors:** Petra Kosutova, Nikolett Nemcova, Maros Kolomaznik, Daniela Mokra, Andrea Calkovska, Pavol Mikolka

**Affiliations:** 1 Biomedical Centre Martin, Jessenius Faculty of Medicine in Martin, Comenius University in Bratislava, 036 01 Martin, Slovakia; 2 Department of Physiology, Jessenius Faculty of Medicine in Martin, Comenius University in Bratislava, 036 01 Martin, Slovakia

**Keywords:** meconium aspiration, surfactant inactivation, oxidative damage, inflammation

## Abstract

Aspirated meconium into a newborn’s airways induces the transcription of pro-oxidative mediators that cooperate in the pathogenesis of inflammatory changes and may negatively affect the commonly used exogenous surfactant therapy. However, inflammation is not treated at present, nor is the time dependence of oxidative damage known. The aim of our study was to describe the time course of oxidative stress marker production during meconium aspiration syndrome (MAS) and its relationship to leukocyte infiltration. New Zealand rabbits were instilled with saline or meconium suspension and ventilated for 5.5 h. Respiratory parameters were recorded and blood samples were taken before meconium application and in time intervals of 15 and 30 min, 1.0, 1.5, 3.5 and 5.5 h after application to evaluate oxidative markers and differential leukocytes count. Meconium aspiration led to a worsening of respiratory parameters and a decrease in leukocytes in the first 15 min. Changes in leukocytes were correlated both with nitrotyrosine (3NT) levels and thiobarbituric acid reactive substance (TBARS) levels, with the latter also related to changes in neutrophil count. The production of 3NT and TBARS increased in 1.5 and 3.5 h, respectively, in different ways, suggesting more than one source of oxidative agents and a potential risk of exogenous surfactant inactivation in a short time. We observed that MAS triggered neutrophil migration to the alveolar space and activation, as shown by the increased expression of pro-inflammatory cytokines and generation of indicators of oxidative damage to proteins and lipids during the time period when iNOS and NO metabolites were released.

## 1. Introduction

Meconium aspiration syndrome (MAS) is defined as respiratory distress due to the presence of meconium in the trachea and is reported to contribute to severe respiratory failure and even death, which is commonly developed during the perinatal and neonatal period [1,2].

Meconium, a newborn’s first stool, is aseptic and contains harmful substances such as bile acids, bile salts, bilirubin, cholesterol, tri-, di-, and monoglycerides, free fatty acids, heme, enzymes such as pancreatic phospholipase A2, and cytokines [3,4,5].

The pathological mechanism of MAS is extremely complex. Multiple factors contribute to the injury of pulmonary vascular endothelial cells and alveolar epithelial cells, such as neutrophil proliferation, activation, and chemotaxis; the elevation of reactive oxygen species (ROS) and protease levels; or activation of inflammatory signaling pathways, which contributes to the promotion of pulmonary microvascular permeability. Subsequently, numerous liquids containing proteins and fibrous proteins infiltrate the pulmonary mesenchyme and alveoli, resulting in noncardiogenic pulmonary oedema and a hyaline membrane, as well as pulmonary mesenchymal fibrosis [6,7,8].

Thus, being germ-free, meconium is able to induce intracellular cascades similar to those induced by bacterial infection. The CD14/TLR4/MD-2 complex can be found in different types of cells, including macrophages, endothelial and epithelial cells [9,10,11,12] and binding of its ligand leads to an increase in the level of transcription factor nuclear factor κB (NF-κB) level. NF-κB translocation to the nucleus simultaneously initiates both pro-oxidative and inflammatory cascades that are interlinked and considered complementary components of inflammatory response [12,13]. The pro-oxidative part of TLR4-induced inflammation lies in the activation of enzymes capable of producing reactive oxygen and nitrogen species (RONS)—inducible nitric oxide synthase (iNOS) [13] and NADPH oxidase (NADPHox) [14]. Excess free radicals damage surfactant proteins and lipids, attack tight junctions facilitating extravasation, and initiate apoptotic signalization in the cells [15,16]. The production of inflammatory factors, the activation of iNOS in macrophages and endothelial cells, and the secretion of nitric oxide (NO) are significantly induced by oxidative stress under acute lung injury (ALI) conditions, which further contributes to the production of stronger oxidants and the development of oxidative injury [17,18,19].

The inflammatory cascade is initiated by the action of cytokines. Meconium is believed to be a powerful stimulator of inflammatory mediators, including cytokines (IL-1β, IL-6, IL-8, and TNFα), complement, prostaglandins, and reactive oxygen species (ROS), according to the majority of available research [5]. Moreover, many of the above-mentioned cytokines are produced in macrophages, endothelial and epithelial cells after TLR4 stimulation [11,20].

As a consequence of the abundance of chemotactic IL-8, massive migration of neutrophils was observed into lung tissue. Once activated, neutrophils harm tissue in both lines—the production of NADPHox- and iNOS-derived radicals worsens the oxidative load while degranulation and release of proteolytic enzymes degrade surfactant components, cell–cell adhesions, and cells themselves [21].

Due to the complexity and interactions of its signal cascades, inflammation does not appear at the very beginning of MAS, but develops gradually over time. Thus, the inactivation of exogenously added surfactant can arise later in the course of time, which may explain the phenomenon of “post-surfactant slump”, sometimes seen in clinical praxis [22]. Therefore, there is a strong need to further examine meconium-induced neutrophil migration and oxidative damage in the timely periods after aspiration.

In the present study, our objective was to compare the time response pattern of derived oxidative and nitrosative stress, white blood cells in peripheral blood, and respiratory and blood gas parameters in meconium-induced lung injury.

## 2. Methods

### 2.1. Animals and Treatment

Meconium aspiration syndrome was experimentally induced by intratracheal instillation of meconium suspension in the lungs of adult rabbits of both genders. Meconium had been obtained from 20 healthy-term newborns, lyophilized and stored at −20 °C until day of use. Immediately before the experiment, the meconium powder was resuspended in 37 °C saline to reach a concentration of 25 mg/mL in a dose of 4 mL/kg b.w. divided into two equal portions while the animals were positioned to the right and left. The experimental design was identical to that used in our previous study [23] with slight modifications being mentioned below and was approved by the Ethics Committee of the Jessenius Faculty of Medicine and by the Slovak Republic’s State Veterinary and Food Executive.

Briefly, after intramuscular anesthesia, the animals were intubated and catheterized in the femoral artery and right atrium for blood sampling and in the femoral vein for continued application of anesthetics. Animals were ventilated with room air for the registration of the values of the basic parameters and then saline or meconium was administered. From this moment on, the animals were ventilated with volume control with a positive end-expiratory pressure (PEEP) of 0.5 kPa, tidal volume (V_T_) of 6 mL/kg, inspiration expiration rate (I:E) of 1:2, respiratory rate (RR) of 40 breaths per minute (bpm), and inspiratory oxygen fraction FiO_2_ of 1.0 throughout the experiment for 5.5 h. For the experiments, 18 New Zealand white rabbits with a body weight of up to 2.5 kg were used. The animals were randomly assigned to the saline group (n = 9, Sal) and the meconium group (n = 9, Mec).

### 2.2. Blood Gases and Ventilation Parameters

Blood gases and oxygen saturation in arterial blood (SaO_2_) were analyzed by Rapidlab (Bayer Diagnostics, Germany) before the administration of saline/meconium (basal value—BV) and 0.5, 1, 1.5, 2.5, 3.5, 4.5 and 5.5 h after administration; partial pressure of oxygen in the femoral artery (PaO_2_) to the fraction of inspired oxygen (FiO_2_) as the P/F ratio and the oxygenation index (OI) = (mean airway pressure × FiO_2_) /PaO_2_ were calculated.

### 2.3. Arterial Blood Sample Collection and Processing

Blood samples were taken from the femoral artery prior to saline/meconium administration (BV) and at the time of 15 and 30 min, 1, 1.5, 3.5 and 5.5 h after administration. The total leukocyte count was determined in a Bürker chamber after Türck staining and the differential leukocyte count was estimated microscopically after Pappensheim staining. After centrifugation at 1000× *g*/4 °C for 15 min, plasma was obtained and stored at −80 °C for further analysis.

### 2.4. Bronchoalveolar Lavage of the Lungs Post Mortem

At the end of the experiment, the animals were killed by an overdose of anesthetics, and the lungs and trachea were excised. The left lungs were lavaged three times using 10 mL of saline (37 °C) per kg of body weight. The bronchoalveolar lavage fluid (BALF) was centrifuged at 600× *g* for 15 min and the differential leukocyte count was evaluated microscopically in sediment after Pappensheim staining. The supernatant was stored at −80 °C until further analysis.

### 2.5. Pulsating Bubble Surfactometer Sample Preparation

Surfactant was isolated from rabbit BALF by taking the supernatant from 15 min, 600× *g* spin and centrifuged 65 min at 40,000× *g* at 4 °C. The surfactant pellet of this high-speed spin was resuspended in 0.5 mL of saline and the amount of phospholipids was quantitated. Surface activity was assessed on a pulsating bubble surfactometer at a final phospholipid concentration of 1.5 mg/mL [24].

### 2.6. Lung Tissue Homogenates

Lung tissue from the non-lavaged part was cut into small pieces and homogenized in a homogenization buffer (30 mM KH_2_PO_4_, 5 mM EDTA, 0.3 M sucrose, pH 7.0, together with 0.3 mM phenyl methyl sulfonyl fluoride (PMSF)) by the Potter-Elvehjem homogenizer, or stored in RNA stabilization solution RNA*later* (QIAGEN Group, Hilden, Germany) until after real-time PCR analysis.

### 2.7. Determination of Oxidative Stress Parameters

The levels of 3-nitrotyrosine (3NT); the concentration of reactive substances of thiobarbituric acid (TBARS), total antioxidant capacity (TAC) and nitrate/nitrite in plasma; and lung tissue homogenates were determined. Products of oxidative stress were evaluated using an OxiSelect™ Nitrotyrosine ELISA Kit (STA-305; Cell Biolabs Inc., San Diego, CA, USA) and an OxiSelect™ TBARS Assay Kit (STA-330; Cell Biolabs Inc., San Diego, CA, USA), and Cayman’s Nitrate/Nitrite Colorimetric Assay Kit (Alexis Corp., San Diego, CA, USA), and OxiSelect™ Total Antioxidant Capacity Assay Kit (STA-360; Cell Biolabs Inc., San Diego, CA, USA) kits were evaluated according to the manufacturer’s instructions.

### 2.8. mRNA Expression Using Quantitative PCR

Stabilized lung tissue was homogenized for 20 s at the maximum speed of the Polytron PT 1200 E homogenizer (Kinematica AG, Malters, Switzerland) and isolated using the RNeasy^®^ Mini Kit (QIAGEN Group, Hilden, Germany). An amount of 1 µg of total mRNA in the reaction was used to produce complementary DNA (cDNA) using a random initiator QuantiTect^®^ reverse transcription kit (QIAGEN Group, Hilden, Germany). The primer sequences are listed in Table 1. Hypoxanthine phosphoribosyl transferase (HPRT) was used as a reference normalized gene. Quantitative real-time PCR (qPCR) was performed with the QuantiTect^®^ SYBR^®^ Green PCR Kit (QIAGEN Group, Hilden, Germany) using the iCycler iQ^®^5 (Bio-Rad Laboratories, Inc., Hercules, CA, USA) for 45 cycles at 95 °C for 15 s, followed by primer-specific annealing temperature at 60 °C for 1 min and 72 °C for 30 s. The crossing point, or the cycle number at which the fluorescence of the sample exceeded that of the background, was determined by Bio-Rad iQ5—Standard Edition Optical System Software 2.0 using the second derivative method. All qPCR analyzes were performed in triplicates.

### 2.9. Evaluation of Surface Activity

The surface activity of the pulmonary/endogenous surfactant was evaluated on a pulsating-bubble surfactometer (PBS; General Transco Inc., Seminole, FL, USA). Approximately 40 µL of pulmonary surfactant was filled into the acrylic sample chamber placed in PBS and heated to 37 °C. A bubble of radius 0.4 mm was generated and pulsated at a rate of 20 cycles/min (radius between 0.4 and 0.55 mm, corresponding to 50% expansion and compression of the area). The pressure across the bubble wall was continuously recorded digitally within 300 s of pulsation. Values of surface tension (minimum bubble size; γmin) were calculated according to the Laplace equation [25].

### 2.10. Statistical Evaluations

For the concentration of oxidative stress products and the total leukocytes count, the results are expressed as a percentage of BV. Regarding the differential leukocytes count, changes in the relative count of neutrophils of BV have been calculated for each sample. Data are expressed as mean ± standard deviation (SD). The effect of the group and the time point of blood sampling was evaluated using two-way analysis of variance (ANOVA) followed by Duncan’s post hoc test; differences between the different groups were analyzed using Mann–Whitney test and correlations were evaluated using the Spearman rank order test.

## 3. Results

### 3.1. Respiratory Parameters

There were no significant differences between the experimental groups in lung function parameters for the baseline values (BV) (Sal versus Mec for all parameters *p* > 0.05). Significant deterioration in the ratio of arterial oxygen partial pressure to inspired oxygen fraction (P/F), oxygenation index (OI), mean alveolar pressure (MAP), and oxygen saturation in arterial blood (SaO_2_) was observed immediately 0.5 h after meconium instillation in the Mec group compared to the control saline group; for P/F and OI *p* < 0.01, for MAP and SaO_2_ *p <* 0.05. This deteriorated effect persisted throughout the experiment (Table 2).

### 3.2. Nitrotyrosine Production

**Figure 1:**
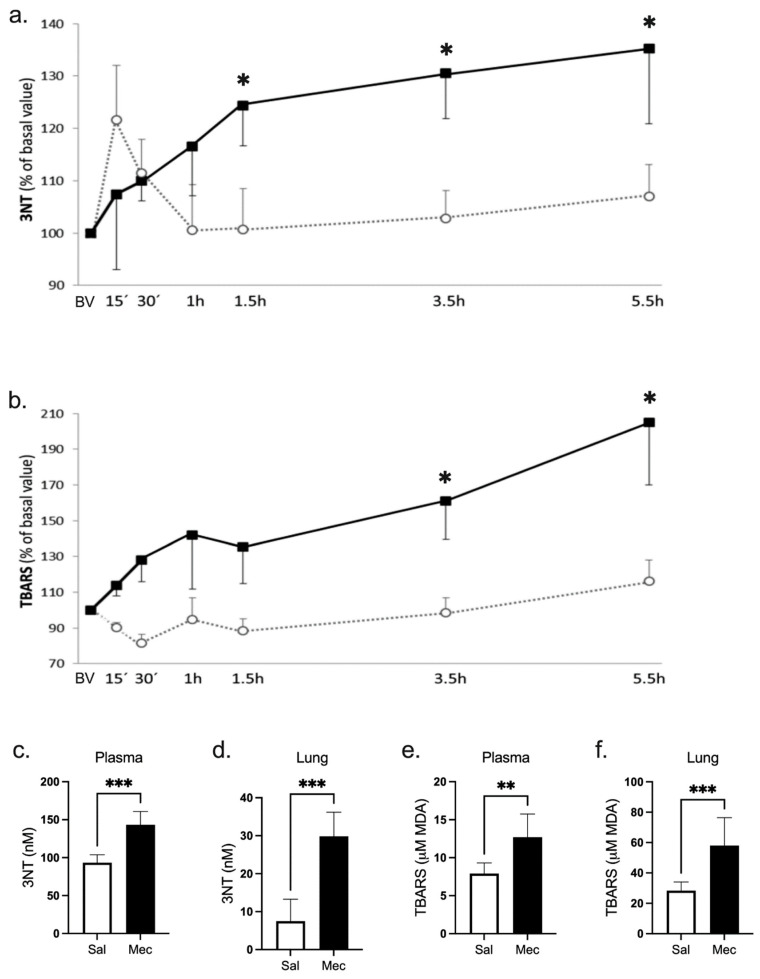
Nitrotyrosine and TBARS Production. (**a**) Nitrotyrosine levels (3NT, nM) and (**b**) reactive substances with thiobarbituric acid reactive substances (TBARS in μM MDA) during experiment; 3NT and TBARS in final plasma (**c**,**e**) and in lung tissue (**d**,**f**) in the saline group (open circles) and the meconium group (closed squares). Statistical comparisons for Mec vs. Sal * *p <* 0.05, ** *p <* 0.01, *** *p <* 0.001.

Intratracheal meconium instillation led to a significant increase in 3-nitrotyrosine (3NT) concentration in the 1.5 h interval compared to the BV of the meconium-treated group. Similarly, the difference in 3NT levels between the Mec and Sal groups reached significance at the same time with persistence to the end (*p <* 0.05) (Figure 1a). During the first 15 min of the experiment in the Sal group, we observed a transient but not significant elevation (*p* = 0.1 vs. Mec) of 3NT levels. Similarly, the 3NT concentration measured in the final plasma and lung tissue was significantly higher in the Mec group compared to the Sal group (for both *p <* 0.001) (Figure 1c,d).

### 3.3. Reactive Substances of Thiobarbituric Acid Production

Unlike oxidative damage to proteins, the impairment of lipids was not evident before 3.5 h after meconium instillation (compared to BV), while the difference between the groups was borderline (Mec vs. Sal *p* = 0.08 from 30 min to 1.5 h). Interestingly, the level of reactive substances of thiobarbituric acid (TBARS) increased continuously during the experiment and the value in 5.5 h was significantly higher than that in 1.5 h (*p <* 0.01) and borderline higher (*p* = 0.06) than in 3.5 h. Such a tendency was not seen in 3NT formation. When comparing experimental groups, a significant difference was observed at 3.5 and 5.5 h of observation (both p < 0.05) (Figure 1b). Furthermore, TBARS levels in final plasma (*p <* 0.01) and lung tissue (*p <* 0.001) significantly increased in the Mec group compared to the saline-treated group (Figure 1e,f).

### 3.4. Changes in the NO Pathway

**Figure 2:**
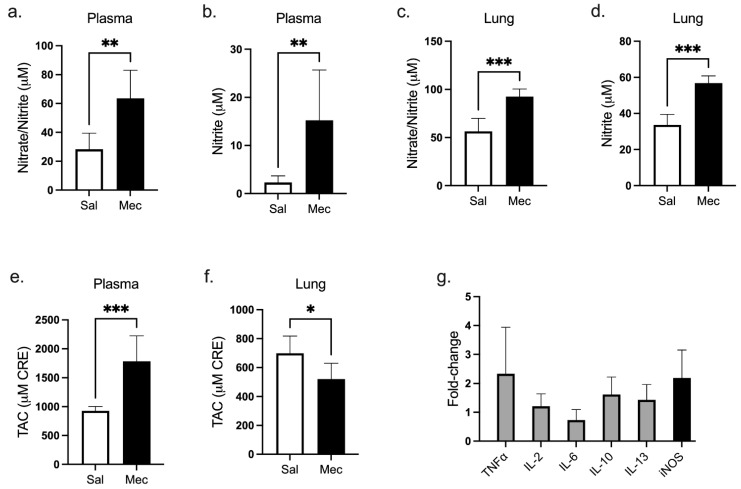
Changes in the NO pathway. (**a**–**d**) Plasma and lung tissue concentration of nitrate/nitrite (μM); (**e**,**f**) total antioxidant capacity (TAC, μM CRE) in plasma and lung, and (**g**) cytokine and iNOS mRNA expression in lung tissue represented as a fold change. The gene expression in the saline group represents the base line (zero). Statistical comparisons for Mec vs. Sal * *p <* 0.05, ** *p <* 0.01, *** *p <* 0.001.

Meconium instillation resulted in a significant increase in nitrite/nitrate and nitrite levels in final plasma (both *p <* 0.01) and lung tissue (both *p <* 0.001) compared to the group treated with saline (Figure 2a–d). Furthermore, in the homogenized lung tissue of Mec animals, we found a decrease in total antioxidant capacity (TAC, *p* < 0.05 vs. Sal), but, on the contrary, a significant increase in TAC in plasma compared to the saline group (Figure 2e,f). For the evaluation of a relative change in iNOS mRNA expression in lung tissue, the saline group was used as a reference group with the expression of the iNOS gene taken as a base value 0. The expression of iNOS after meconium instillation in the Mec group increased approximately twofold compared to that of the saline group. Furthermore, we observed an increase in the expression of interleukins in the Mec group compared to saline (Figure 2g).

### 3.5. Total and Differential Leukocyte Count in Plasma and BALF

**Figure 3:**
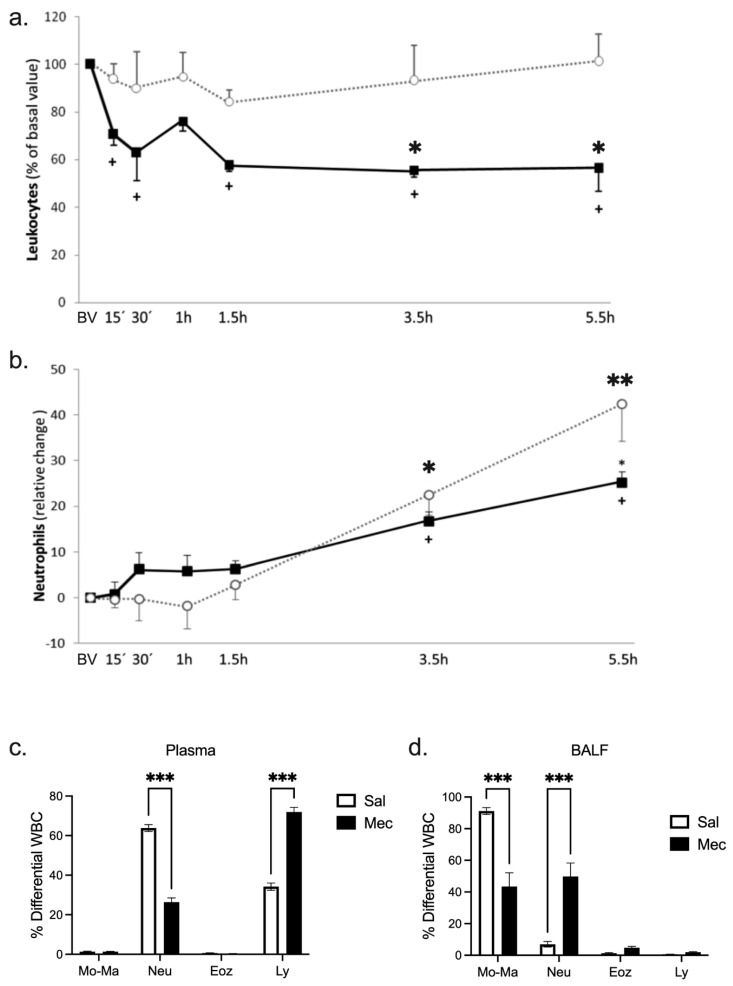
Total and differential leukocyte count. (**a**) Leukocyte and (**b**) neutrophil changes in blood in the saline group (open circles) and the meconium group (closed squares); (**c**,**d**) percentage of differential leukocytes in arterial blood and BALF. Abbreviations: neutrophils (Neut), lymphocytes (Ly), macrophage monocytes (Mo-Ma), eosinophils (Eoz). Statistical comparisons for Mec vs. Sal * *p <* 0.05, ** *p <* 0.01, *** *p <* 0.001; + *p <* 0.05 vs. meconium basal value (BV).

In plasma, meconium aspiration led in the first 15 min to a significant decrease in total leukocyte count compared to the baseline value (BV) that lasted throughout the experiment (*p <* 0.05) except for the time point of 1 h. A decrease in leukocytes count was more pronounced in the Mec group and this difference reached statistical significance after 3.5 h of the experiment compared to the Sal group; 30 min *p* = 0.06, 1.5 h *p* = 0.07, 3.5 and 5.5 h *p <* 0.05 (Figure 3a). As a consequence of demargination, the relative count of neutrophils increased during the experiment in both groups (*p <* 0.05 vs. BV for both groups in 3.5 and 5.5 h). However, this increase was lower in the Mec group, which was reflected in significant differences compared to the Sal group (Figure 3b). The percentage of lymphocytes decreased in both groups, markedly in the Sal group, reflecting the significant difference observed compared to the Mec group (Figure 3c). In BALF, infiltrated neutrophils were markedly present in animals instilled with meconium compared to the saline group (*p <* 0.001), which affected a significant decrease in the percentage of monocyte macrophages (Mec vs. Sal *p <* 0.001) (Figure 3d).

### 3.6. Correlations

We observed a significant relationship between 3NT and TBARS production and the number of leukocytes in arterial blood (for 3NT *r* = −0.36, *p <* 0.01, for TBARS *r* = −0.41, *p <* 0.001). Similarly, the correlation of neutrophil changes with TBARS production reached statistical significance (*r* = 0.27, *p <* 0.05), while there was no relationship between neutrophil count and 3NT levels.

### 3.7. Surface Activity of the Pulmonary/Endogenous Surfactant

**Figure 4:**
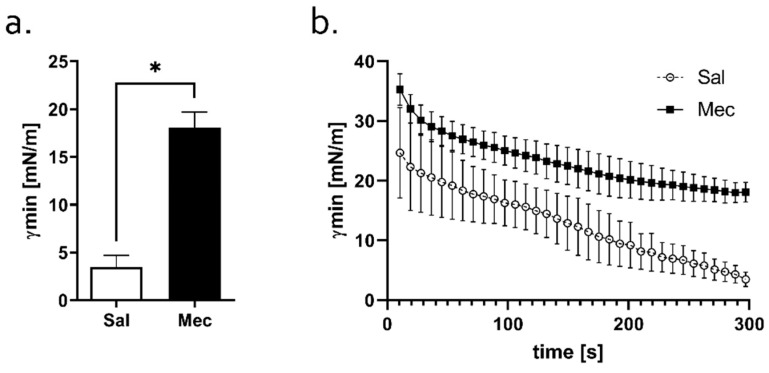
Surface activity of the surfactant obtained from BALF. (**a**) Minimum surface tension (ST; γmin) after 300 s of pulsation using the pulsation bubble surfactometer (PBS), (**b**) minimum ST of the pulmonary surfactants during the entire analyzed period for 300 s of cycling in PBS. Statistical comparisons for Mec vs. Sal * *p <* 0.05.

After 300 s of pulsation in PBS, γmin of the Mec group pulmonary/endogenous surfactant was significantly higher compared to the Sal group surfactant (*p*  *<*  0.05) (Figure 4a). The dynamic changes in γmin of both pulmonary/endogenous surfactants throughout the period (300 s) of cycling in PBS are shown in Figure 4b.

## 4. Discussion

In this study, we provide detailed time responses to oxidative and nitrosative stress and changes in leukocyte count in the first minutes after experimental induction of MAS. Meconium inactivates the pulmonary surfactant, triggers oxidation and inflammation, and damages the endothelial and epithelial cells. In experimental animals with meconium instillation, similar symptoms can be observed that express a significant deterioration in lung function. Meconium decreases lung compliance by 40–50% and worsens gas exchange within 30 min after instillation [23,26,27]. Meconium caused a significant increase in the deterioration of all respiratory and blood gas parameters (P/F, OI, MAP, PaCO_2_, SaO_2_) immediately after administration compared to the control group, which is consistent with the findings of other studies [23,28,29,30]. A mismatch in ventilation/perfusion in the Mec group was characterized by a decrease in oxygen saturation to 80% when OI exceeded 10.

Meconium can chemoattract neutrophils, monocytes, and macrophages and trigger their leakage through the alveolocapillary membrane [31,32,33]. Activated cells in lung tissue produce numerous potentially harmful substances that cause inflammatory and oxidative and nitrosative changes. Cytokines and chemokines secreted by macrophages and polymorphonuclears, such as IL-1β, IL-8, and TNF-α, stimulate further neutrophil transmigration, amplify the inflammatory cascade, and aggravate lung injury [34,35]. Activated inflammatory cells also produce excessive concentrations of RONS, proteases, etc., which exacerbate capillary permeability and hemorrhage, and extend the time of neutrophil sequestration [36]. Considering the role of inflammation and oxidative damage in MAS, the objective of this study was to investigate meconium-induced neutrophil migration and oxidative damage in the period after aspiration. We found that MAS induced neutrophil migration to the alveolar space and caused their activation, as verified by the increased expression of pro-inflammatory cytokines and production of markers of oxidative damage to proteins and lipids during the period of time expression of iNOS and NO metabolites. Our findings are consistent with previous studies [23,29,37].

Inflammation is associated with an increase in pro-inflammatory cytokines, and it increases the production of free oxygen radicals via the induction of oxidative stress [38]. There is the assumption that meconium itself and also the meconium-induced production of free radicals and inflammatory mediators might be able to inactivate endogenous surfactant [16]. The rate of surfactant inactivation depends on the intensity of inflammatory processes that vary significantly over time. Leukocytes synthesize and release inflammatory mediators, including nitric oxide (NO) [39,40]. Inducible NO synthase (iNOS) is induced by inflammatory-like stimuli and can produce large amounts of NO that predominate during inflammation [41]. It is assumed that meconium activates TLR4 in different cell types, initiating cascades that lead to NF-κB activation and its translocation to the nucleus [42,43]. As a consequence, the expression of high-output iNOS is set off [44]*,* and a variety of cytokines, chemokines, and adhesion molecules are produced even in those cells which, by definition, does not belong to the immune system [42,45]. While cytokines and adhesion molecules, among other things, assert in neutrophil migration, high NO release facilitates peroxynitrite formation. Peroxynitrite, being a strong oxidant, rapidly attacks proteins at tyrosine residues, which can be seen as an increase in nitrotyrosine levels [46]. NF-B activation of NF-κB in macrophages in the presence of meconium became prominent after 30 min and the concentration of nitrite as an end product of NO formation in the medium-incubated cells increased by 4 h [47]. However, when assessing iNOS activation in acute in vivo experiments, we found an increased degree of protein nitrosylation already 1.5 h after meconium treatment, suggesting that under these conditions, iNOS was activated more rapidly. NF-κB activation that precedes iNOS induction may be initiated by other molecular pathways independently of TLR4 signalization. It is well-known that NF-κB expression is directly induced by ROS action, derived predominantly from NADPHox [48] or certain cytokines [49] originating from meconium itself and some immune cells. Intercellular cooperation during inflammatory cascades in the context of ROS signalization has been extensively addressed but is still poorly understood [45]. It has been shown that TBARSs and nitrate/nitrite are part of the most important components of oxidative stress after inflammation, and increased levels of TBARSs and nitrite/nitrate have been found in lung injury [50,51,52,53,54,55]. We detected a significantly increased level of TBARSs 3.5 h after meconium administration in plasma, and this increased level was observed until the end of the experiment compared to the control group. We observed an increased level of TBARSs in lung tissue in meconium-induced lung injury. During our experiment, after meconium administration, TBARS and 3NT production tended to rise continuously in plasma, and we also observed significantly increased levels of these oxidative markers in lung tissue compared to controls. An imbalance in increased levels of oxidants and impaired antioxidant induction has been documented in experimental ALI models and in ARDS patients [56,57,58,59,60]. Antioxidants are the primary defense against RONS. In the present study, we chose to detect TAC in lung tissues and plasma. Our data showed that the TAC in the Mec group was significantly lower in lung tissue, but in plasma, we observed a significantly increased TAC level in the Mec group. The antioxidant activities were markedly decreased, indicating an oxidant/antioxidant imbalance that favors oxidative stress in the lungs and leads to lung injury [61].

Activated neutrophils are markedly increased in lung tissue during MAS [29]. We demonstrated the migration process already 15 min after meconium administration, similar to the previous study [62]. The rapid influx of demarginated neutrophils after meconium instillation appears to be caused by multiple factors, including the presence of chemoattractive IL-8 in meconium [5], its production in lung tissue after TLR4 activation [20], and also NF-κB-induced production of adhesive molecules that allow neutrophil migration through the capillary wall [11,63]. Furthermore, damage to intercellular junctions due to free radicals and proteinases released by formerly activated neutrophils simplifies the influx of other polymorphonuclears [64]. The announced fall in neutrophil count in peripheral blood and its proportional increase in BALF after 5.5 h of experiment suggest that in our model of MAS, chemoattractive stimuli—and that also means inflammation magnitude—are strong.

The correlation analysis revealed a strong relationship between oxidative damage and leukocyte migration to the lungs. Changes in neutrophil count were connected with TBARS production, but there was no correlation with 3NT formation. We suggest that protein nitrosylation does not need to presumably be a consequence of neutrophil-derived iNOS action more than that from other cells. iNOS may also be expressed in macrophages and some nonimmune cells such as endothelial cells and epithelial cells [65] as all of those mentioned possess TLR4 receptors on their surface and so may contribute to oxidative impairment. Moreover, endothelial cells possess an endothelial isoform of NOS which, under conditions of oxidative stress, can simultaneously produce both NO and superoxide radical [65,66,67,68]. The important conclusion remains that protein and lipid damage—even surfactant ones—occurs during MAS in a relatively short period of time, arises out of more sources and exceeds tissue antioxidant abilities.

Surfactant dysfunction caused by meconium is a multilevel process that begins with the direct effect of meconium on surfactant components, followed by harmful inflammatory and oxidative cascades that are harmful [69,70]. The components of meconium are toxic to lung tissue and induce an inflammatory response and oxidative damage. The impairment of any of the surfactant components may affect biophysical activity and contribute to altered lung function in patients with MAS [71,72]. Therefore, we also focused on the evaluation of the surfactant activity of the pulmonary/endogenous surfactant obtained from rabbit bronchoalveolar lavage fluid. In our experiments, the administration of meconium increased γmin, that indicated impaired surface activity of the pulmonary surfactant. In addition, the inflammation and oxidative stress associated with MAS, together with plasma proteins and meconium itself, affect the structure of the surfactant and increase its minimum surface tension [71].

## 5. Conclusions

In conclusion, inflammation and oxidation associated with MAS present a very complex response of many types of cells communicating and cooperating at the level of signal molecules. In this study, we observed that oxidative and nitrosative stress were significantly activated in plasma and lung tissues, which was consistent with the description in previous reports [18,37]. Despite the several limitations, e.g., using young adults instead of rabbit puppies, lung injury induction by a single event, and several inter-species differences in the innate immune response, data from this study may be useful at least for a better understanding of the pathophysiology of MAS [73]. In addition, observation of time-dependent changes may serve as a good starting point for targeted anti-inflammatory and antioxidative stress therapy to treat MAS, but further research in this field is necessary.

## Figures and Tables

**Table 1:** Sequences of primers.

Gene	Primer	Sequence
HPRT	Forward Reverse	5′-TGATAGATCCATTCCTATGACTGTAGA-3′ 5′-GGGTCCTTTTCACCAGCAG-3′
IL-2	Forward Reverse	5′-TGAAACATCTTCAGTGTCTAGAAG-3′ 5′-CATTGTAGAATTTCTGAACAGAT-3′
IL-6	Forward Reverse	5′-TAGTCCTTCCTACCCAATTTCC-3′ 5′-TTGGTCCTTAGCCACTCCTTC-3′
IL-13	Forward Reverse	5′-GCAAATAATGAGCTTTCGAAGTTTCAGTGG-3′ 5′-CTTCCGTGAGGACTGAATGAGACGGTC-3′
IL-10	Forward Reverse	5′-GAGAACCACAGTCCAGCCAT-3′ 5′-CATGGCTTTGTAGACGCCTT-3′
TNF α	Forward Reverse	5′-GTCTTCCTCTCTCACGCACC-3′ 5′-TGGGCTAGAGGCTTGTCACT-3′
iNOS	Forward Reverse	5′-GCAGCAGCGGCTTCACA-3′ 5′-ACATCCAAACAGGAGCGTCAT-3′

**Table 2:** Respiratory parameters. The ratio of partial pressure of arterial oxygen to inspired oxygen fraction (P/F, kPa), oxygenation index (OI), mean alveolar pressure (MAP, kPa), partial pressure of carbon dioxide (PaCO_2_, kPa), oxygen saturation in arterial blood (SaO_2_, %) and pH during the experiment (basal value, BV) in the saline (Sal) and meconium (Mec) groups. Statistical comparisons for Mec vs. Sal * *p <* 0.05, ** *p <* 0.01, *** *p <* 0.001.

	BV	0.5 h	1.5 h	3.5 h	5.5 h
**P/F (kPa)**					
Sal	39.6 ± 2.3	30.9 ± 6.7	51.9 ± 4.9	54.9 ± 1.9	47.6 ± 4.0
Mec	42.3 ± 2.6	6.9 ± 0.6 **	10.4 ± 2.7 ***	7.6 ± 0.5 ***	7.0 ± 0.7 ***
**OI**					
Sal	0.8 ± 0.1	4.3 ± 0.6	1.4 ± 0.1	1.3 ± 0.1	1.3 ± 0.1
Mec	0.7 ± 0.1	13.1 ± 0.8 **	11.0 ± 1.4 ***	12.4 ± 0.7 ***	14.1 ± 1.2 ***
**MAP (kPa)**					
Sal	0.3 ± 0.0	0.7 ± 0.1	0.6 ± 0.1	0.6 ± 0.1	0.6 ± 0.1
Mec	0.3 ± 0.0	1.0 ± 0.0 *	1.0 ± 0.0 *	1.0 ± 0.0 *	1.0 ± 0.0 *
**PaCO_2_ (kPa)**					
Sal	5.2 ± 1.3	6.4 ± 1.2	5.6 ± 1.0	5.9 ± 1.0	6.2 ± 1.1
Mec	5.1 ± 1.1	10.6 ± 1.8 *	8.5 ± 2.2	9.4 ± 1.2 *	11.5 ± 2.5 *
**SaO_2_ (%)**					
Sal	92.3 ± 4.7	96.9 ± 2.3	99.7 ± 0.1	99.7 ± 0.2	99.7 ± 0.1
Mec	89.8 ± 3.1	79.6 ± 2.2 *	82.6 ± 3.2 *	77.2 ± 3.3 **	75.1 ± 4.8 **
**pH**					
Sal	7.5 ± 0.1	7.3 ± 0.1	7.3 ± 0.0	7.3 ± 0.0	7.2 ± 0.1
Mec	7.5 ± 0.1	7.2 ± 0.1	7.2 ± 0.1	7.1 ± 0.1 *	7.0 ± 0.0 *

## Data Availability

The data are available on request from corresponding author.
